# Modulation of Leukocyte Subsets Mobilization in Response to Exercise by Water Immersion Recovery

**DOI:** 10.3389/fphys.2022.867362

**Published:** 2022-08-16

**Authors:** Vinícius de Oliveira Ottone, Fabrício De Paula, Paula Fernandes Aguiar Brozinga, Mariana Aguiar de Matos, Tamiris Campos Duarte, Karine Beatriz Costa, Bruna Caroline Chaves Garcia, Thyago José Silva, Flavio De Castro Magalhães, Cândido Celso Coimbra, Elizabethe Adriana Esteves, Kelerson Mauro de Castro Pinto, Fabiano Trigueiro Amorim, Etel Rocha-Vieira

**Affiliations:** ^1^ Exercise Biology and Immunometabolism Laboratory, Centro Integrado de Pós-graduação e Pesquisa em Saúde, Graduate Program in Physiological Sciences, Universidade Federal dos Vales do Jequitinhonha e Mucuri, Diamantina, Brazil; ^2^ Faculty of Medicine, Universidade Federal dos Vales do Jequitinhonha e Mucuri, Diamantina, Brazil; ^3^ Faculdade de Sete Lagoas, Sete Lagoas, Brazil; ^4^ Graduate Program on Health Sciences, Universidade Federal dos Vales do Jequitinhonha e Mucuri, Diamantina, Brazil; ^5^ Departament of Physical Education, Faculty of Biological and Health Sciences, Universidade Federal dos Vales do Jequitinhonha e Mucuri, Diamantina, Brazil; ^6^ Departament of Physiology and Biophysics, Faculty of Biological Sciences, Universidade Federal de Minas Gerais, Belo Horizonte, Brazil; ^7^ Departament of Nutrition, Faculty of Biological and Health Sciences, Universidade Federal dos Vales do Jequitinhonha e Mucuri, Diamantina, Brazil; ^8^ School of Physical Education, Universidade Federal de Ouro Preto, Ouro Preto, Brazil; ^9^ Exercise Physiology Laboratory, Department of Health, Exercise and Sport Science, University of New Mexico, Albuquerque, NM, United States

**Keywords:** hot-water immersion, non-classical monocytes, acute inflammation, CD25, lymphocyte, endurance exercise, resistance exercise

## Abstract

**Purpose:** To investigate the effect of different water immersion temperatures on the kinetics of blood markers of skeletal muscle damage and the main leukocyte subpopulations.

**Methods:** Eleven recreationally trained young men participated in four experimental sessions consisting of unilateral eccentric knee flexion and 90 min of treadmill running at 70% of peak oxygen uptake, followed by 15 min of water immersion recovery at 15, 28 or 38°C. In the control condition participants remained seated at room temperature. Four hours after exercise recovery, participants completed a performance test. Blood samples were obtained before and immediately after exercise, after immersion, immediately before and after the performance test and 24 h after exercise. The number of leukocyte populations and the percentage of lymphocyte and monocytes subsets, as well as the serum activity of creatine kinase and aspartate aminotransferase were determined.

**Results:** Leukocytosis and increase in blood markers of skeletal muscle damage were observed after the exercise. Magnitude effect analysis indicated that post-exercise hot-water immersion likely reduced the exercise-induced lymphocytosis and monocytosis. Despite reduced monocyte count, recovery by 38°C immersion, as well as 28°C, likely increased the percentage of non-classical monocytes in the blood. The percentage of CD25^+^ cells in the CD4 T cell subpopulation was possibly lower after immersion in water at 28 and 15°C. No effect of recovery by water immersion was observed for serum levels of creatine kinase and aspartate aminotransferase.

**Conclusions:** Recovery by hot-water immersion likely attenuated the leukocytosis and increased the mobilization of non-classical monocytes induced by a single session of exercise combining resistance and endurance exercises, despite no effect of water immersion on markers of skeletal muscle damage. The monocyte response mediated by hot water immersion may lead to the improvement of the inflammatory response evoked by exercise in the skeletal muscle.

## 1 Introduction

A local, acute inflammatory response is associated with long duration, unaccustomed exercise, and eccentric muscle action (Peake et al., 2017; Cerqueira et al., 2020; Markus et al., 2021). This response is activated by exercise-induced tissue disturbance (i.e., cell damage/stress and protein leakage) and the release of stress signals into the circulation. This leads to the trafficking of inflammatory cells to the exercised musculature where leukocytes initiate a repair response, that may be also associated with the trigger of signals involved in the adaptive response of the skeletal muscle to exercise (Pizza et al., 2002; Suzuki et al., 2002; Chazaud et al., 2009; Tidball and Villalta, 2010; Paulsen et al., 2012). However, depending on its quality, magnitude and duration, the inflammatory response can be detrimental to tissue physiology (Carvalho et al., 2010; Tidball and Villalta, 2010; Puntel et al., 2011). In the context of the exercised muscle, inflammation has been also implicated in increased muscle soreness, edema, and decreased muscle function, leading to reduced performance capacity and fatigue commonly experienced following various forms of strenuous exercise (Smith, 1991; Byrne and Eston, 2002; Cheung, Hume and Maxwell, 2003).

With the aim to accelerate exercise recovery, many strategies are employed by athletes, recreational exercisers, and the general population. Water immersion, specifically at cold temperatures ( ≤  20°C), is one of the most popular methods used to improve post exercise recovery (Versey, Halson and Dawson, 2013; Dupuy et al., 2018; Ahokas et al., 2020), although immersion protocols vary in terms of water temperature (hot water, 36°C; thermoneutral water, 21–35°C) (Craig and Dvorak, 1966) and depth, immersion duration, and frequency.

Several mechanisms have been suggested to account for the enhanced acute recovery associated with post-exercise water immersion, and they are primarily associated with hydrostatic pressure and water temperature effects. Hydrostatic pressure during thermoneutral immersion increases fluid displacement from the periphery toward the central cavity. The resulting increased stroke volume, total peripheral vascular resistance, and cardiac output can potentially enhance oxygen and metabolite transport (Johansen et al., 1997; Wilcock, Cronin and Hing, 2006a). These alterations likely assist recovery due to enhanced removal of metabolic waste products from the working muscles and reduced transport time of oxygen and nutrients to the exercised muscles (Nakamura et al., 1996; Wilcock, Cronin and Hing, 2006b). Hydrostatic pressure can also promote fluid shifts between tissue compartments and limit edema formation and swelling (Mishra et al., 1995; Wilcock, Cronin and Hing, 2006a) which in turn maintains muscle oxygen delivery and contractile function. Moreover, hydrostatic pressure exerts a buoyant force that reduces neuromuscular activation (Pöyhönen et al., 1999; Pöyhönen and Avela, 2002) potentially facilitating relaxation of postural muscles and contributing to the reduction of the perception of fatigue (Nakamura et al., 1996; Kuligowski et al., 1998).

These effects are influenced by water temperature. Cold water immersion, for example, decreases skin, core, and muscle temperatures (Peiffer et al., 2009, 2010; Gregson et al., 2011), which can account for improved performance in long duration tests, especially in warm/hot temperatures, due to a pre-cooling effect (Quod, Martin and Laursen, 2006; Xu et al., 2021). Cold water immersion is also associated with vasoconstriction, which further increases central blood flow (Choo et al., 2018) and promotes a relative decrease in the local blood flow (Fiscus, Kaminski and Powers, 2005; Vaile et al., 2011; Seeley, Giersch and Charkoudian, 2021). Thus, this can lead to theoretical reduction of swelling and edema. Muscle pain can be also reduced by cold water immersion due to the reduction of nerve conduction velocity (Abramson et al., 1966; Washington, Gibson and Helme, 2000).

Hot water immersion is associated with higher heart rate and cardiac output, compared to thermoneutral immersion. Increased superficial temperature and peripheral vasodilation are also observed (Myrer et al., 1997; Fiscus, Kaminski and Powers, 2005; Vaile et al., 2011). Cutaneous and subcutaneous blood flow are increased by hot water immersion, due to increased cardiac output and lower peripheral resistance, which increases vascular permeability. This can result in increased nutrient delivery and waste removal from tissues, although it is argued that these effects are more likely to take place in the skin rather than the exercised muscle. A slow decrease in core temperature after exercise is also found in hot water immersion (Vaile et al., 2008). If muscle temperature is elevated during subsequent exercise it may enhance muscle function and performance (Fradkin, Zazryn and Smoliga, 2010). The effect of hot water immersion on neurotransmission, proprioception and reaction time is also proposed, although research-based evidence to support it is still lacking (Versey, Halson and Dawson, 2013).

A potential anti-inflammatory effect has been also attributed to recovery by water immersion and, it is used to attenuate the detrimental effects of post-exercise inflammation. This is grounded, for example, in the water hydrostatic pressure effect on edema formation (Mishra et al., 1995; Wilcock, Cronin and Hing, 2006a) and in the vasoconstriction induced by cold water immersion, which can potentially attenuate inflammatory signaling and cell trafficking to the stressed muscle (reviewed by Ihsan, Watson and Abbiss, 2016). Also, the reduced tissue temperature following cold water immersion reduces O_2_ consumption (Yanagisawa et al., 2004; Yanagisawa, Takahashi and Fukubayashi, 2010), which in turn would reduce local metabolic stress and further reduce the release of inflammatory signals from the muscle (Merrick et al., 1999). On the other hand, heat application can exacerbate swelling and inflammation (Johansen et al., 1997).

The evidence to support water immersion as a modulator of exercise-induced stress and inflammation remains equivocal. While many studies suggest that water immersion is not effective in reducing cellular stress and inflammation (Peake et al., 2017), others suggest that exposure to cold may enhance the acute inflammatory response to exercise (Stacey et al., 2010; White, Rhind and Wells, 2014; Wilson et al., 2018, 2019) and even that it can be blunted by thermoneutral or cold-water immersion (Pournot et al., 2011; Earp et al., 2019). It can be argued that the diversity of markers employed in many studies can account for these discrepancies. Although this is a factor to be considered, opposite or no effects of water-immersion interventions are still observed, even when the same markers are considered. When creatine kinase (CK) leakage is considered for example, some reports indicate that hot or cold water immersion prevents its increase after exercise (Ascensão et al., 2011; Minett et al., 2014). Others observed no effect of thermoneutral or cold-water immersion on serum CK activity (Sellwood et al., 2007; Goodall and Howatson, 2008; Ahokas et al., 2019), or increased CK activity after recovery by hot water immersion (Viitasalo et al., 1995). Data on the benefits of water immersion methods on exercise-induced leukocytosis are also inconsistent, with studies demonstrating no effect of cold-water immersion on the count of blood leukocyte populations (Pournot et al., 2011; de Andrade Bezerra et al., 2014; Ahokas et al., 2020) or greater leukocyte and neutrophil mobilization and reduced lymphocyte counts 1 h after exercise followed by cold-water immersion (Stacey et al., 2010). Data on hot water immersion on cell stress and inflammation are still lacking, and the scarce studies available suggest no effect on the post-exercise leukocyte response (Pournot et al., 2011). The failure to include appropriate control conditions and different study designs can account for these conflict findings as well. Additionally, so far, no study investigated whether leukocyte subpopulations (i.e., T helper and cytotoxic and B lymphocytes, NK cells, monocytes subsets) are affected by exercise recovery by water immersion in different temperatures and how this is correlated to skeletal muscle stress/damage markers.

Therefore, in this study, using a crossover-controlled design, we investigated the effectiveness of 3 water immersion interventions (cold, hot and thermoneutral water), compared to a no-water recovery control, on the kinetics of blood markers of muscle damage and main leukocyte populations and subpopulations. Different water temperatures were chosen because the physiological response elicited by immersion per se can be modified or potentiated by water temperature, and different temperatures can trigger additional effects, also influencing exercise recovery (Vaile et al., 2011; Peake et al., 2017; Choo et al., 2018; Ahokas et al., 2020). The exercise and recovery protocols were designed to match athletes’ training routines. Thus, the exercise sessions were composed of both endurance and resistance exercises. Also, athletes often engage in two exercise sessions per day (i.e., morning and afternoon) during training periods and may have multiple competitive events separated by several hours. In this context, a second exercise session at the end of day, approximately 4 h after the first, was employed to investigate whether athletes would benefit from any recovery strategy in this circumstance. We hypothesized, based on the effects of water immersion on vascular permeability, that cold-water would reduce and hot-water immersion would increase the post-exercise leakage of skeletal muscle proteins and leukocytosis.

## 2 Materials and Methods

### 2.1 Participants and Ethical Statement

The present study enrolled eleven non-smokers, healthy and trained young men (54.5 ± 3.5 ml kg^−1^ min^−1^ peak oxygen uptake; 24.0 ± 5.0 years-old; 178.0 ± 7.0 cm height; 73.0 ± 13.3 kg of body mass; 10.8 ± 3.5% of body fat). Participants were not professional athletes regularly engaged in various intermittent activities such as soccer, cycling and running, 2 to 5 times a week, each session lasting over 2 h. Inclusion criteria required that participants have a peak oxygen uptake (VO_2_peak) > 50 ml kg^−1^ min^−1^. Prior to data collection, a medical questionnaire was completed by the participants to exclude individuals who were taking medications or had recent musculoskeletal injuries. The physical activity readiness questionnaire (PAR-q) (Thomas, Reading and Shephard, 1992) was used to exclude individuals at risk for performing physical activity.

All subjects were informed of the experimental procedures and the risks associated with participation and signed an informed consent form. The study was approved by the Internal Human Research Ethics Committee Review Board (# 057/2010).

### 2.2 Study Design

Participants performed an exercise protocol (experimental exercise) consisting of localized lower limb eccentric and prolonged moderate intensity running exercises, followed by 15 min of recovery by immersion at water in different temperatures, in a crossover-controlled design. Four hours later, participants were submitted to a performance test. Blood markers of skeletal muscle damage, as well as leukocyte populations and lymphocyte and monocyte subpopulations were monitored throughout the experimental period, until 24 h after the experimental exercise. Physiological (heart rate, heart rate variability, rectal temperature, excessive post-exercise oxygen consumption) and performance data, obtained in this same occasion, had been previously published (De Oliveira Ottone et al., 2014; De Paula et al., 2018).

### 2.3 Anthropometrics, Preliminary Evaluations, and Familiarization

Prior to the experimental trials, participants went through 3 preliminary sessions. In the first session, body fat percent was calculated using the sum of seven site skin folds (Jackson and Pollock, 1978). During this session, all subjects completed a maximal graded exercise test, using a treadmill ramp protocol test to determine individual VO_2_peak. The VO_2_ peak was measured by indirect calorimetry (K4b2, Cosmed) using a customized ramp protocol, performed on a treadmill (PRO 300 RT, Movement, Brazil), as described elsewhere (De Oliveira Ottone et al., 2014). After a 3 min warm-up at 5 km h^−1^, the treadmill speed and grade were increased every 60 s, until volitional fatigue, despite verbal encouragement. The speed increments were based on training history and was designed to fatigue participants at between 8 and 12 min of running. Heart rate (HR) was recorded during the test using a telemetric HR transmitter strap and watch (S810i series TM, Polar, United States). To meet the criteria for VO_2_ peak, the subject had to attain at least two of the following criteria: a respiratory equivalent ratio greater than 1.15, a HR within 5% of age-predicted maximal HR (220-age), or a plateau of oxygen consumption (≤150 mLO_2_ kg^−1^ min^−1^) with increase in work intensity (Yoon, Kravitz and Robergs, 2007).

In the second visit to the laboratory, leg extensor strength through dynamic contraction was assessed by measuring the maximum amount of weight lifted for one repetition (one-repetition maximum–1RM test) using a seated knee extension machine (Master, Brasil). The test was conducted as previously described (De Paula et al., 2018). Subjects first warmed-up by completing 5 to 10 repetitions at 40–60% of their estimated 1RM. The 1RM was measured in four to five trials by successively increasing the load for each lift, with rest periods of 2–3 min between each trial. Standard verbal encouragement was used during the 1RM test. After the 1RM test, the participants rested for 30–40 min, and then completed a 5-km self-paced familiarization run (aerobic performance test), as previously described (De Paula et al., 2018).

In the third preliminary visit, volunteers performed the 5-km time trial test, in which participants completed a self-paced 5-km time trial. No information about running speed or time was provided to the participants, although they were informed about the distance covered at each kilometer.

All exercise sessions were conducted in a controlled, temperate environment (20.0 ± 2.0°C and 70.0 ± 10.0% relative humidity). Volunteers arrived at the laboratory in the morning (∼8:00 a.m.). They were previously instructed to refrain from exhaustive exercise, alcohol, and caffeine consumption during the previous 24 h. They were also asked to ingest 500 ml of water 2 h before arriving at the laboratory.

### 2.4 The Experimental Sessions

**FIGURE 1:**
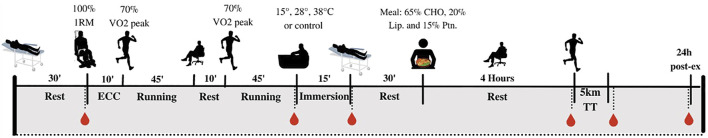
Overview of the study design.

The study timeline is illustrated at Figure 1. At least 36 h after the last preliminary session, each participant was required to complete 4 crossover experimental sessions, separated by at least 5 days (7 ±  2 days). At the laboratory, volunteers rested for 30 min in the supine position before completing the experimental exercise session, composed of resistance exercise and submaximal running. In each experimental trial, the exercise session was followed by one of the four recovery strategies, for 15 min: no immersion (CON), immersion in hot (38°C), temperate (28°C) or cold water (15°C). For each group of 4 participants, the subjects started with a different immersion condition, and the following sessions occurred in the order illustrated in Table 1, to avoid or minimize repeated bout effects. Thereafter, the subjects recovered at room temperature for 240 min before the aerobic performance test, after which they were discharged from the laboratory.

**TABLE 1:** Cross-over design used in the study.

Participants	Experimental Conditions			
	CON	38°C	28°C	15°C
A	1st	2nd	3rd	4th
B	4th	1st	2nd	3rd
C	3rd	4th	1st	2nd
D	2nd	3rd	4th	1st
E	1st	2nd	3rd	4th
F	4th	1st	2nd	3rd
G	3rd	4th	1st	2nd
H	2nd	3rd	4th	1st
I	1st	2nd	3rd	4th
J	4th	1st	2nd	3rd
K	3rd	4th	1st	2nd

The eleven participants of the study (A to K) were allocated, in each group of 4, to initiate the study in a different recovery condition, and, from the CON, condition, the sequence of the subsequent protocols was always the same.

#### 2.4.1 The Experimental Exercise

The experimental exercise session was composed of eccentric exercise followed by submaximal running. The eccentric exercise consisted of 3 series of 10 repetitions of unilateral knee flexion for both legs, at 100% of 1RM, measured during the concentric phase. Researchers moved participants’ leg through the concentric phase (knee extension) and subjects performed only the eccentric phase (knee flexion). This resistance exercise session lasted approximately 10 min. Immediately after this, volunteers completed a prolonged, moderate intensity, continuous running session on a motorized treadmill: 2 bouts of 45 min, at 70% of VO_2_ peak, with 10 min of rest between bouts. Oxygen consumption was measured in the first experimental session for each participant, and the adjustments made in the treadmill speed in that first session was matched for the remaining three sessions.

Immediately after the experimental exercise, volunteers were assigned to 15 min of recovery by water immersion at 15°, 28° or 38°C, or control (CON), followed by 30 min of rest. Subjects then received a standard meal composed of 65% carbohydrates, 20% lipids, and 15% proteins, based on their energy expenditure and dehydration during the exercise. Energy expenditure was determined based on the metabolic equivalents corresponding to the exercise intensity. Water was provided to replace the difference in body weight pre- and post-exercise. Subjects remained at rest in the laboratory, awake, for a period of 4 h, prior to the 5-km time trial. They were allowed to read, watch movies, and access the internet, but not to leave the laboratory or to be physically active.

Before the experimental session, participants were instructed to refrain from exercise, alcohol, and caffeine consumption during the previous 24 h. All volunteers recorded their food intake on the night prior to and at the day of the first experimental session and were asked to repeat their ingestion for the following experimental sessions. The successful replication of the food intake was checked upon the arrival of the subject at the laboratory on each experimental day. They were also asked to drink 500 ml of water 2 h before arriving at the laboratory. Hydration status of the volunteers was checked by the evaluation of urine specific gravity (handheld clinical refractometer, model 301, Biobrix, Brazil). Participants with urine specific gravity higher than 1.030 (Armstrong et al., 1998; Oppliger et al., 2005) were considered dehydrated and the experimental session was scheduled for another day.

All sessions were conducted in a controlled temperate environment (19.8 ± 1.8°C and 71.0 ± 9.0% relative humidity).

#### 2.4.2 Post-Exercise Recovery

Immediately after the experimental exercise session, the volunteers were assigned to passive recovery for 15 min, in either water immersion at 15, 28 or 38°C ( ± 1°C) or control (CON) with no immersion. During water immersion, volunteers were seated (20° of knee flexion) in a plastic drum and submersed to the xiphoid process level, with the arms out of the water, wearing only shorts. Water temperature was achieved using a thermal resistance (38 and 28°C) or by means of crushed ice (15°C). Water was circulated every 3 min to maintain a uniform temperature, and verified with a digital thermometer (YSI 4600, Ohio, United States). These 3 water temperatures were chosen to change the rectal temperature while remaining tolerable to the volunteers. In the CON condition, participants remained seated in the plastic drum for 15 min, without water. Immediately after the initial 15 min recovery volunteers were dried and rested in supine position, covered in a blanket for an additional 30 min.

#### 2.4.3 Aerobic Performance Test

The self-paced 5-km time trial occurred 4 h following the end of water immersion or control recovery. No information about speed or time was provided to the participants during the time trial. Volunteers were discharged from the laboratory after the test.

### 2.5 Blood Collection and Analysis

Blood samples were collected from the participants, via an intravenous catheter (22 × 0.9 mm), attached to a 3-way stopcock, inserted on the antecubital vein, during the following time points: before the experimental exercise (pre-ex), immediately after the experimental exercise (post-ex), 15 min after the recovery (post-im), immediately before and after the aerobic performance test (pre-t and post-t) and 24 h after the experimental exercise (24 h post-ex). Blood samples were collected in EDTA tubes, for blood leukocyte populations and subpopulations quantification, and dry tubes for the determination of serum creatine kinase (CK) and aspartate transaminase (AST) activities.

Acute shifts in plasma volume due to exercise were calculated using the equation proposed by Dill and Costill, 1974.

#### 2.5.1 Serum Activity of Aspartate Transaminase and Creatine Kinase

A blood sample (approximately 1 ml) was centrifuged (400 g, 15 min, 25°C) and the plasma was collected for the measurement of AST and CK. The measurement of AST activity was carried out by monitoring the rate of NADH oxidation, in a coupled reaction system, employing malate dehydrogenase, using a commercially available kit (Transaminase AST cinética, K048, Bioclin, Brazil) and a semi-automatic analyzer (PW-3000M, Pioway, China), as previously described (Tossige-Gomes et al., 2016). The plasma CK activity was also determined by an UV kinetic method, using a commercially available kit (CK NAC UV K010, Bioclin, Brazil). This method monitors the rate of NADP^+^ reduction, at 340 nm, in a coupled reaction system employing hexokinase and glucose 6-phospate dehydrogenase. Under circumstances in which CK activity is rate limiting, the rate of reduction of NADP^+^ to NADPH is directly proportional to the enzyme concentration in the sample.

#### 2.5.2 Quantification of Blood Leukocyte Populations and Subpopulations

Total and differential leukocyte counts (neutrophil, lymphocyte, monocyte, eosinophil and basophil) were performed using a Celm^®^ CC530/550 Analyzer (Celm, Brazil) and Giemsa’ stained blood smears.

Flow cytometry was used to determine the percentage of lymphocyte (CD4 and CD8 T cells, CD25CD4 T cells, B, NK and NKT cells) and monocyte (classical—CD14^++^CD16^−^, intermediate—CD14^++^CD16^+^ and non-classical CD14^+^CD16^++^) subsets, using 50 μL of whole blood, as previously described (de Matos et al., 2016; Tossige-Gomes et al., 2016). Cells were stained with monoclonal antibodies conjugated to specific fluorochromes [mouse anti-human CD3-FITC or -PerCP (clone HIT3a), CD4-PE (clone RPA-T4), CD8-PerCP (clone MOPC-21), CD25-FITC (clone M-A251), CD19-PE (clone HIB19), CD16-FITC (clone 3G8), CD14-PerCP (clone MφP9); CD56-PE (clone B159)]. The antibodies were all branded BD Pharmingen (San Diego, CA, United States).

The data were acquired (30,000 total events) in the FACSCan flow cytometer (Becton & Dickinson, Franklin Lakes, NJ, United States). Cell Quest (Becton & Dickinson) software was used for data analysis. Lymphocytes and monocytes were identified according to their distribution profile in forward (FSC) versus side scatter (SSC) dot plots. From events in the lymphocyte and monocyte gates, the percentages of CD3^+^CD4^+^, CD3^+^CD8^+^, CD3^+^CD4^+^CD25^+^, CD19^+^, CD3^−^CD16^+^ CD56^+^, CD3^+^CD16^+^ CD56^+^ and CD14^+^CD16^−^, CD14^+^CD16^+^ and CD14^+^CD16^++^ cells were determined. Non-stained, isotype and fluorescence minus one (FMO) controls were used to establish gate and/or quadrant positioning.

### 2.6 Statistical Analysis

GraphPad Prism (version 8.00 for Mac OS X, GraphPad Software, San Diego, CA, United States) was used for statistical analysis. Data are reported as mean ± SD. The Shapiro–Wilk test was used to assess the normality of the data. Mauchly’s test of sphericity assessed data sphericity, and the Greenhouse-Geisser correction was applied when the assumption of sphericity was violated. Two-way repeated-measures analysis of variance (ANOVA) was used to compare continuous variables between conditions (immersion at different temperatures versus CON) across pre- and post-exercise time points. Due to two missing data points in the leukocyte subset analyses, a mixed effects model using the maximum likelihood method was performed instead of a two-way repeated-measures ANOVA. The Dunnet’s test, with correction for multiple comparisons using statistical hypothesis testing, was used as post-hoc to explore time effects within trials, compared to pre-exercise. One-way repeated-measures ANOVA, followed by Dunnet’s test, was used to compare rectal temperature variation in the different water temperature conditions to CON. Statistical significance was set at *p* ≤ 0.05.

Additionally, to within-trial changes comparison (between recovery conditions effect), qualitative analyses were conducted using magnitude-based inferences, as proposed by (Hopkins, 2006). The smallest worthwhile change/difference was calculated as 0.2 multiplied by the between subject standard deviation, according to Cohen’s effect size principle (Cohen, 1988), to analyze the potential trends in leukocytes subsets during each timepoint compared with CON. Data are expressed as percent chances of a high, trivial, or low outcome. Quantitative chances of higher or lower differences were ranked as follows: <1%, almost certainly not; 1–5%, very unlikely; 5–25%, unlikely; 25–75%, possibly; 75–95%, likely; 95–99%, very likely; >99%, almost certain.

## 3 Results

In this study we investigated the effect of post-exercise recovery by immersion in water at different temperatures on inflammatory markers. The internal (rectal) temperature was increased by 1.7 ± 0.4°C at the end of the exercise, and it was reduced by 1.2 ± 0.4°C during the 15 min control (CON) condition, compared to the post-ex value. The repeated one-way ANOVA revealed that recovery of rectal temperature was modified by water immersion (treatment main effect, F = 19.21, *p* < 0.001, η^2^ = 0.71), according to the temperature of the water: it was reduced by 1.5 ± 0.5°C after cold-water immersion (*p* = 0.02, compared to CON) and, after hot water immersion, rectal temperature reduced only 0.7 ± 0.5°C, compared to post-ex value (*p* = 0.0004, compared to CON). A 1.1 ± 0.6°C reduction in rectal temperature was observed after immersion in thermoneutral water, compared to post-ex (*p* = 0.99, power = 0.96), compared to CON). Our data show that core temperature recovery was accelerated by cold-water immersion and delayed by immersion at hot water.

As shown in Table 2, the repeated two-way ANOVA revealed that the serum activity of both AST and CK were increased after the exercise and throughout the experimental period (main exercise effect, F = 23.37 and 15.31, *p* = 0.0004 and 0.002, η^2^ = 0.79 and 0.72, AST and CK, respectively). This indicates the protocol employed was of sufficient intensity to promote skeletal muscle cell disturbance and protein leakage. No effect of recovery by water immersion was observed on the serum activity of these enzymes, at any timepoint evaluated.

**TABLE 2:** Effect of post exercise recovery by water immersion on muscular damage markers.

	Pre-ex	Post-ex*	Post-im*	Pre-t*	Post-t*	24 h Post-ex*
AST activity						
CON	28.1 ± 5.6	34.8 ± 9.1	32.3 ± 8.2	34.2 ± 8.8	46.4 ± 10.4	44.7 ± 17.5
38°C	27.7 ± 5.7	30.5 ± 9.4	30.4 ± 7.6	33.2 ± 9.9	37.8 ± 12.7	42.6 ± 8.5
28°C	27.9 ± 5.3	32.3 ± 7.0	32.8 ± 6.3	32.2 ± 6.7	43.7 ± 11.4	45.0 ± 10.0
15°C	26.7 ± 6.1	30.6 ± 5.3	28.9 ± 11.3	32.5 ± 4.5	35.5 ± 6.9	47.1 ± 10.2
CK activity						
CON	298.7 ± 193.2	437.5 ± 221.0	426.6 ± 232.4	471.6 ± 241.5	539.4 ± 263.0	614.9 ± 414.6
38°C	236.9 ± 156.0	372.8 ± 180.2	394.6 ± 224.1	398.8 ± 213.7	475.2 ± 247.4	513.9 ± 290.2
28°C	301.5 ± 203.4	372.7 ± 234.9	356.9 ± 155.3	334.0 ± 152.2	431.2 ± 239.6	715.8 ± 153.9
15°C	352.4 ± 232.8	453.6 ± 227.3	446.0 ± 246.3	509.1 ± 232.9	532.4 ± 215.9	1025.8 ± 497.5

*p < 0.05, main time effect, compared to pre-ex, repeated two-way ANOVA, followed by Dunnett’s multiple comparison test.

Pre-ex, before exercise session; post-ex, immediately after exercise session; post-im, immediately post immersion; pre-t, immediately before performance test; post-t, immediately post performance tests; 24 h post-ex, 24 h after the exercise session.

**FIGURE 2:**
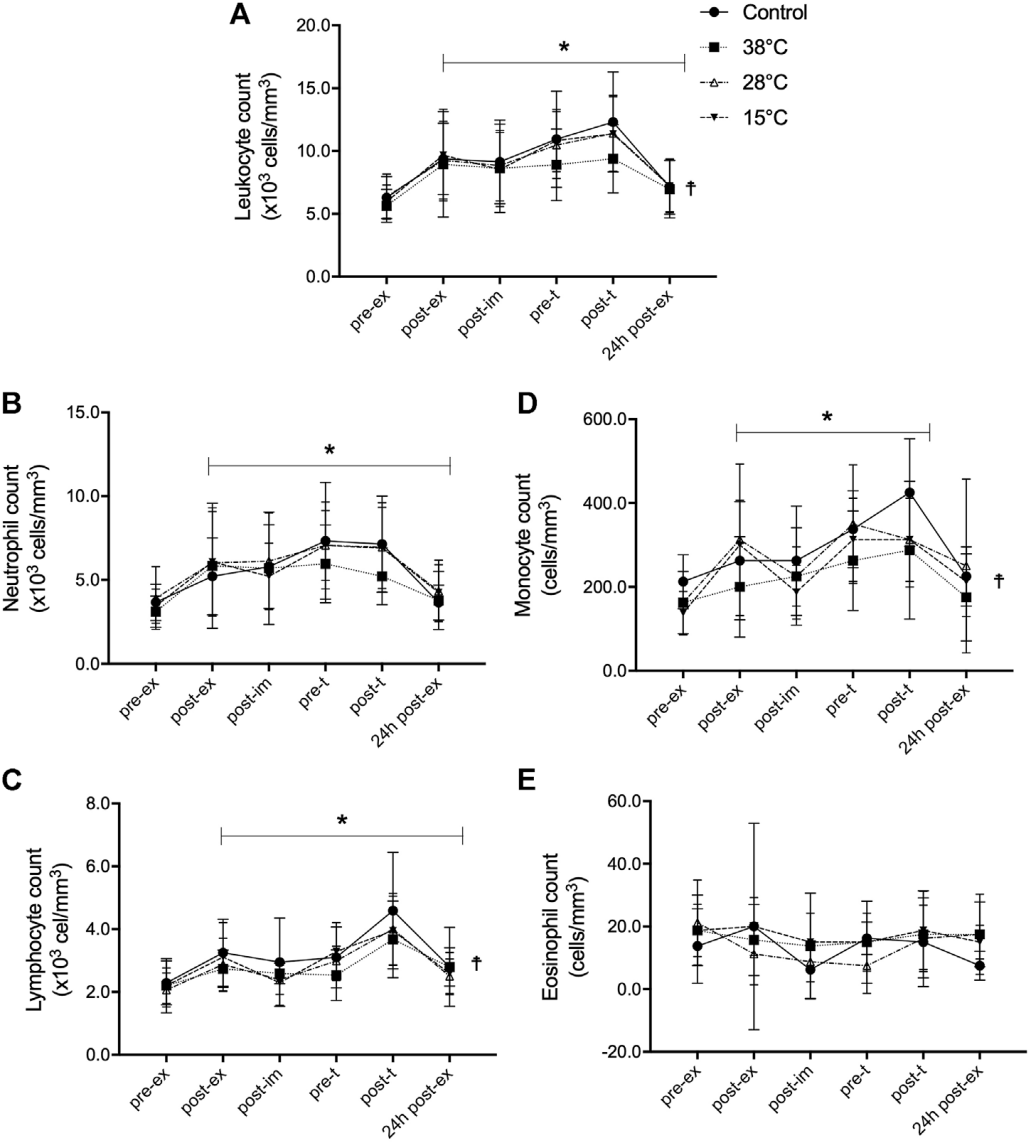
Effect of post-exercise recovery by water immersion on the global and differential leukocyte count. Leukocyte count **(A)**, neutrophil count **(B)**, lymphocyte count **(C)**, monocyte count **(D)**, and eosinhopil count **(E)**. *p < 0.05, main time effect, compared to pre-ex, repeated two-way ANOVA, followed by Dunnett’s multiple comparison test.^☨^ *p* < 0.05, main treatment effect, 38°C versus control, repeated two-way ANOVA, followed by Dunnett’s multiple comparison test. Pre-ex–before exercise session, post-ex–immediately after exercise session, post-im–immediately post immersion, pre-t–immediately before performance tests, post-t–immediately post performance tests, 24 h post-ex–24 h after the exercise session.

The response of blood leukocyte populations to exercise and recovery was also evaluated (Figure 2). An exercise-induced leukocytosis that persisted for 4 h after the exercise (pre-t), was observed (Figure 2A) in the CON condition, as well as in the three water immersion recovery conditions (main exercise effect, F = 17.91, *p* < 0.001, η^2^ = 0.72), as indicated by repeated two-way ANOVA. The leukocytosis was also higher after the performance test (post-t) and remained elevated for 24 h after the exercise in all experimental conditions. A main treatment effect (*p* = 0.03) on leukocytosis was observed, comparing recovery by immersion at 38°C water to the CON.

Neutrophils, lymphocytes, and monocytes all contributed to the observed leukocytosis (Figures 2B–D, respectively). After exercise, the repeated two-way ANOVA revealed that counts of neutrophils (F = 29.14, *p* < 0.001, η^2^ = 0.68) lymphocytes (F = 25.72, *p* < 0.001, η^2^ = 0.73), and monocytes (F = 14.03, *p* < 0.001, η^2^ = 0.65) were greater and they were sustained until 24 h after the exercise, except for the monocyte number (*p* = 0.09, power = 0.12), in all experimental conditions. The neutrophilia induced by exercise was due to the mobilization of both mature segmented and immature band neutrophils (Supplementary Figure S1). A main treatment effect (*p* = 0.01) of recovery was found with immersion in 38°C water on exercise-induced lymphocytosis and monocytosis, but not on the neutrophil count. Neither exercise nor water immersion recovery had any effect on eosinophil (Figure 2E) and basophil counts (data not shown).

Because main treatment effects were observed for leukocyte, lymphocyte and monocyte counts, magnitude-based inference analyzes were conducted (Table 3). Our findings indicate that leukocytosis was likely lower before (pre-t) and after performance tests (post-t) when comparing recovery at 38°C to the CON, with no immersion. This was also observed for the lymphocyte count: a likely lower lymphocyte number was found pre-t and post-t after recovery by hot water immersion, compared to CON. The same was observed for the monocyte number, although this effect was still observed 24 h after the experimental session (24 h post-ex). These findings point to a potential effect of hot water immersion recovery on the exercise induced leukocytosis, via the modulation of lymphocyte and monocyte numbers.

**TABLE 3:** Magnitude-based inferences after recovery by hot- (38°C), thermoneutral- (28°C) and cold- (15°C) water immersion compared to control.

**Cell type**	Intervention Moment			
	post-im	pre-t	post-t	24 h post-ex
Water immersion temperature	Higher/trivial/lower % (qualitative inference)			
Leukocyte				
38°C	7/52/41 (unclear)	3/15/82 (likely)	2/8/90 (likely)	12/57/31 (unclear)
Lymphocyte				
38°C	8/33/59 (unclear)	4/14/82 (likely)	4/17/79 (likely)	20/60/20 (unclear)
Monocyte				
38°C	2/14/84 (likely)	8/11/81 (likely)	2/4/94 (likely)	11/10/79 (likely)
CD25^+^ in CD4 T cells				
28°C	12/33/55 (unclear)	2/26/72 (possibly)	9/22/69 (unclear)	5/23/72 (possibly)
15°C	2/29/69 (possibly)	2/10/88 (likely)	15/43/42 (unclear)	6/26/68 (unclear)
Non-classical monocytes				
38°C	77/20/3 (likely)	38/35/27 (unclear)	93/4/3 (likely)	12/42/46 (unclear)
28°C	87/11/2 (likely)	88/10/2 (likely)	99/1/0 (very likely)	12/30/58 (unclear)

Post-im, immediately post immersion; pre-t, immediately before performance test; post-t, immediately after performance tests; 24 h post-ex, 24 h after the exercise session.

**FIGURE 3:**
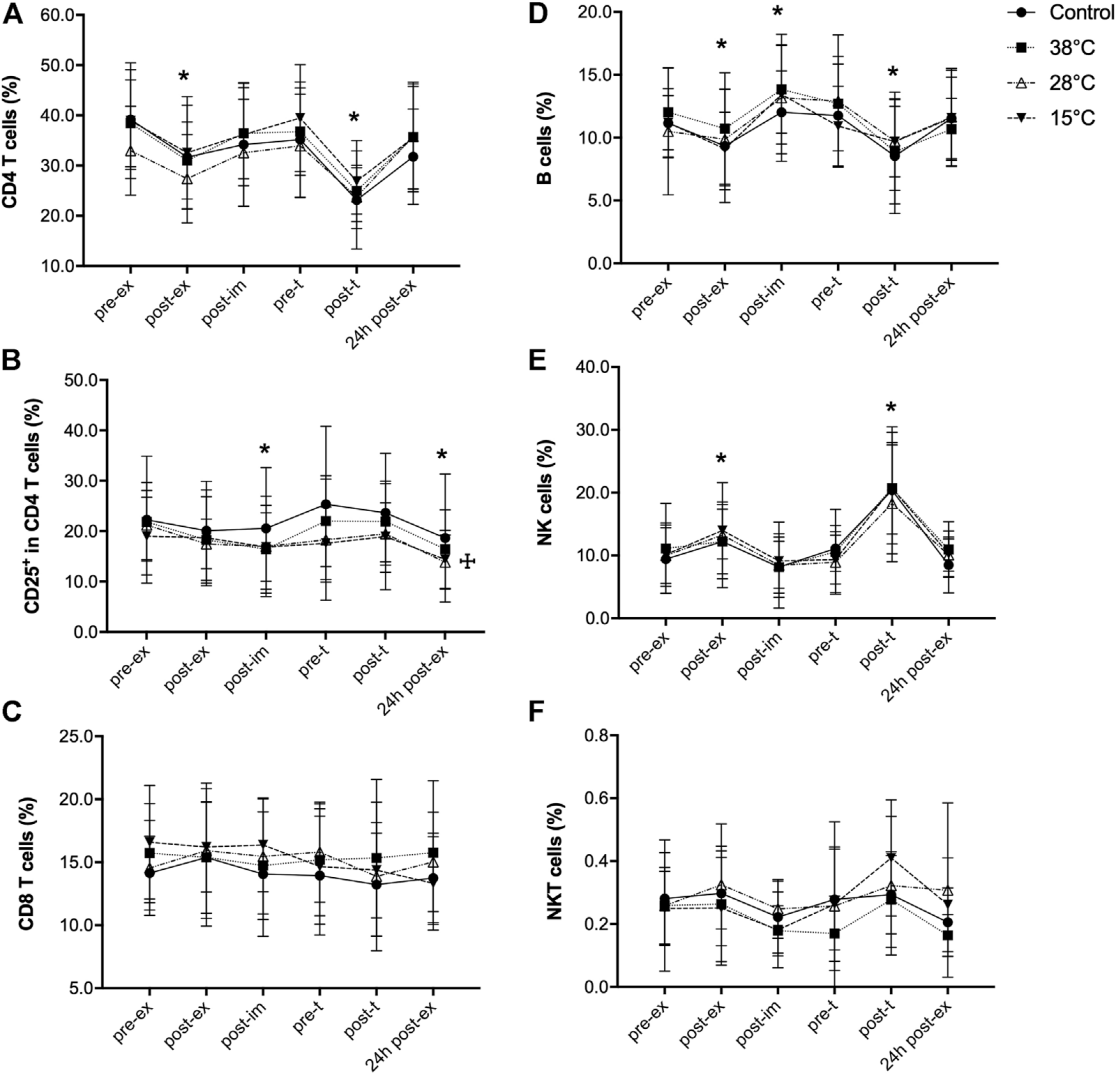
Effect of exercise recovery by water immersion on the percentage of blood lymphocyte subpopulations. CD4 T cells **(A)**, CD25^+^ in CD4 T cells **(B)**, CD8 T cells **(C)**, B cells **(D)**, NK cells **(E)** and NKT cells **(F)**. *p < 0.05, main time effect, compared to pre-ex, mixed-effects analysis, followed by Dunnett’s multiple comparison test. ^**☩**^ *p* < 0.05, main treatment effect, 28°C and 15°C versus control, mixed-effects analysis, followed by Dunnett’s multiple comparison test. Pre-ex–before exercise session, post-ex–immediately after exercise session, post-im–immediately post immersion, pre-t–immediately before performance tests, post-t–immediately post performance tests, 24 h post-ex–24 h after the exercise session.

**FIGURE 4:**
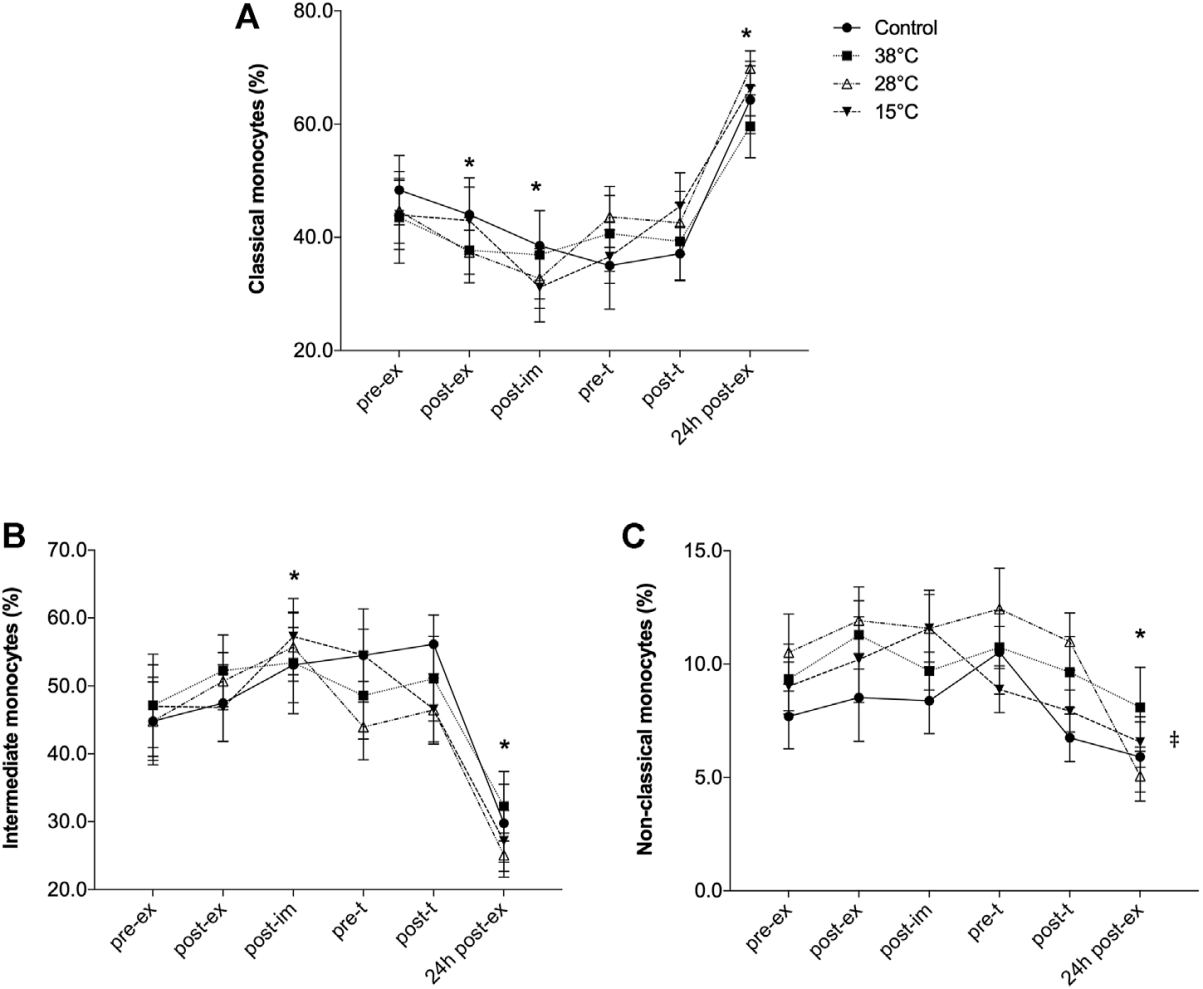
Effect of exercise recovery by water immersion on the percentage of blood monocyte subsets. Classical monocytes **(A)**, Intermediate monocytes **(B)** and Non-classical monocytes **(C)**.*p < 0.05, main time effect, compared to pre-ex, mixed-effects analysis, followed by Dunnett’s multiple comparison test. *p* < 0.05, main treatment effect, 28°C and 38°C versus control, repeated two-way ANOVA, followed by Dunnett’s multiple comparison test. Pre-ex–before exercise session, post-ex–immediately after exercise session, post-im–immediately post immersion, pre-t–immediately before performance tests, post-t–immediately post performance tests, 24 h post-ex–24 h after the exercise session.

We further investigated exercise and water immersion recovery effects on lymphocyte (Figure 3) and monocyte (Figure 4) subpopulations. Due to two missing data points, a mixed effects model was performed instead of a two-way repeated-measures ANOVA, for the subpopulations analysis. Exercise induced a transient, significant reduction in the percentage of CD4 T cells (main exercise effect, F = 19.00, *p* < 0.001), that was quickly recovered after immersion (Figure 3A). This transient, significant reduction in the percentage of CD4 T cells was also observed after the performance test, but it returned to pre-ex values 24 h after the exercise. The percentage of CD4^+^CD25^+^ T cells was also transiently reduced in response to exercise (main exercise effect, F = 14.04, *p* < 0.001) and this reduction was maintained until 24 h after the first exercise session (Supplementary Figure S2). Because these cells are part of the total CD4 T cells, this finding could be due to the reduction in this lymphocyte subpopulation. However, as shown in Figure 3B, the percentage of CD25^+^ cells in the CD4 subpopulation was also reduced in response to exercise (main exercise effect, F = 4.02, *p* = 0.02). This indicates a direct effect of exercise on the distribution of recently activated CD4 T cells in the blood. No effect of water immersion recovery on the CD4 T cells was observed (Figure 3A), although a main treatment effect on CD25CD4 T cells was observed (15 and 28°C versus control) (Figure 3B). No effects of exercise or water immersion recovery were observed on the percentage of CD8 T cells (Figure 3C).

The effect of exercise on the percentage of B cells was different: although B cell percentage was significantly reduced immediately after exercise (main exercise effect, F = 11.01, *p* < 0.001), a transient, significant increase in the percentage of this lymphocyte subpopulation was found 30 min after exercise, with no effect of the recovery method used (Figure 3D). Exercise induced a transient increase of the percentage of NK cells, (main exercise effect, F = 13.6, *p* = 0.0008) with no effect of water immersion recovery (Figure 3E). Neither exercise nor water immersion recovery had any effect on the percentage of NKT cells (Figure 3F).

Exercise and recovery by water immersion also affected the distribution of blood monocyte subsets (Figure 4). The early response of monocytes to exercise was marked by the significant reduction of classical monocytes percentage (main exercise effect, F = 21.33, *p* < 0.001, Figure 4A) and significant increase of intermediate monocytes (main exercise effect, F = 14.77, *p* = 0.0001, Figure 4B). However, 24 h after the exercise session the effect was the opposite: while the percentage of classical monocytes was significantly greater, compared to pre-exercise values the intermediate monocyte percentage was significantly reduced. No effect of the recovery methods on the distribution of these monocytes subsets was observed. However, the percentage of non-classical monocytes was altered only 24 h after the exercise session (F = 5.39, *p* = 0.01) and a main treatment effect was observed (28°C and 38°C versus control, *p* = 0.04).

As indicated in Table 3, the percentage of CD25^+^ cells was possibly and likely lower, post-immersion and before the performance test, respectively, comparing recovery by cold water immersion to CON. Possible lower CD25^+^ cell percentage was also found comparing thermoneutral water immersion to CON before the performance test (pre-t) and 24 h after the experimental exercise session. Non-classical monocytes percentage, however, was likely (post-im and pre-t) and very likely higher (post-t) in response to thermoneutral-water immersion. Also, non-classical monocytes were likely higher after immersion and after the performance test, comparing hot water immersion (38°C) to CON.

## 4 Discussion

This study investigated the effectiveness of three different water immersion temperatures (hot, thermoneutral and cold) on blood leukocyte profile and serum CK and AST activities, after two bouts of exercise, the first one being composed of both endurance and resistance exercises, 4 h apart from each other. Our findings show that hot water immersion (38°C) likely attenuated the exercise-induced leukocytosis, and that the exercise-response of recently activated CD4 T cells and non-classical monocytes were modified according to the water temperature. No effect of water immersion was observed on CK and AST plasma activity.

Post-exercise recovery by water immersion is a method frequently used by athletes, more specifically cold-water immersion, due to its potential anti-inflammatory effect, which would result in accelerated recovery from exercise. Blood leukocytes are feasible markers to monitor the acute inflammatory response to exercise because their response to a myriad of exercises of different mode, duration and intensity is well known (Cerqueira et al., 2020). Also, blood leukocyte counts require low-cost technology, making this an interesting and practical marker to monitor the exercise-induced inflammation in diverse situations. However, investigations about the effect of different water immersion temperatures on post-exercise leukocyte dynamics and studies exploring different leukocyte subpopulations are still lacking. As reported by others (Cerqueira et al., 2020) leukocytosis was found after an experimental exercise session, consisting of eccentric and long-duration endurance exercise. The leukocytosis was due to the increase in the number of neutrophils, lymphocytes and monocytes which was detected even 4 h after the exercise session. Leukocytosis was further increased by the second additional bout of exercise and was noted 24 h after the experimental session. This pattern was also seen for the main leukocyte populations, except for monocytes, whose count returned to baseline 24 h after the exercise session performed on the previous morning. The quantitative statistical analysis indicated a main treatment effect of hot water immersion on the exercise-induced leukocyte response, and magnitude-based analysis showed that leukocyte, lymphocyte and monocyte counts were likely lower after exercise recovery by hot water immersion.

To our knowledge, only one study has been done to investigate the effect of hot water immersion on the response of blood leukocytes to exercise. Pournot et al. (2011)used different protocols, including warm-water immersion (36°C, 15 min, seated with water to the depth of the iliac crest) after two bouts of exhaustive intermittent exercise (countermovement jump and rowing). In the water immersion group the exercise-induced leukocytosis, measured 1 and 24 h after the recovery, was not attenuated, compared to the passive recovery group. It was different from the cold-water immersion (10°C) group, in which leukocytosis in response to the exercise session did not occur. No effect of warm-water immersion was observed on serum CK activity, but cold-water immersion prevented the increase in serum CK, observed in the control group 24 h after the exercise. Exercise mode and duration in the Pornout et al. study are different from the present one, and even the quality of the exercise-induced leukocytosis (no lymphocyte alteration) was different, as well as the post-water immersion time course evaluation. Vaile et al. (2008) also demonstrated the effect of hot water immersion on inflammatory markers, although they did not investigate leukocyte counts. A delayed onset muscle soreness (DOMS)-inducing protocol was used to investigate the effect of hot-water immersion on CK, lactate dehydrogenase, myoglobin and interleukin-6 (IL-6). Recovery consisted of immersion (head and neck out) for 14 min in 38°C water, completed immediately after, and at 24, 48, and 72 h post-exercise. CK, but not the other markers, was reduced 48 h after the exercise by hot-water immersion. In our study we didn’t observe any effect of hot water immersion on the CK response, which can be explained by the moment it was evaluated (24 h post-exercise), and different from (Vaile et al., 2008), we used a single immersion session. Although no alteration in the thigh circumference by hot water was observed in the study of Vaile, assessed by non-stretch measuring tape as an indirect evaluation of post-exercise swelling, it is reasonable to argue whether these findings reflect an attenuation in local leukocyte recruitment.

Another group studied the effect of cold water immersion on the leukocyte response to exercise (Stacey et al., 2010). Participants performed 3 bouts of 50-kJ cycling, as fast as possible, with 20 min of recovery between bouts, 10 min of which immersed on water at 10°C, up to the neck. However, they observed a greater leukocytosis, neutrophilia and lymphopenia 1 h after the recovery by cold-water immersion, compared to rest. Also, no effect of the intervention was observed on IL-6 levels. Similar to our study, the Stacey et al. (2010) study employed a crossover design, while in the study by Pournot et al. (2011) different water temperatures were tested with different groups of individuals. In contrast to these investigations, de Andrade Bezerra et al. (2014) found no effect of recovery by cold-water immersion (10°C, 10 min, immersion to the iliac crest level) on leukocyte count, after a soccer match. Also, no effect was found for leukocyte counts, C-reactive protein and markers of muscular damage after cold- or thermoneutral-water (24°C) immersion following a high-intensity sprint and jumping exercise protocol and active recovery Ahokas et al. (2020). Again, the leukocyte response to the exercise stress in the cited studies is qualitatively different from ours (both Ahokas et al., 2020 and de Andrade Bezerra et al., 2014) observed lymphopenia after exercise), and this leads us to argue whether the effect of water immersion on leukocytes is dependent on the dynamics of the leukocyte response to the exercise. In our study, we did not observe effects of cold- or thermoneutral-water immersion on leukocyte counts, although both water temperatures, beyond 38°C modified the composition of lymphocyte and monocyte subsets after the experimental exercise. The percentage of CD25^+^ cells in the CD4 T cell subpopulation was possibly lower after recovery by cold- and thermoneutral-water immersion. CD25 is the most prominent activation marker of T cells, which is upregulated within 24 h after T cell stimulation, and it remains elevated for a few days (Depper et al., 1983; Hemler et al., 1984). Thus, CD4^+^CD25^+^ T cells are always observed in the peripheral blood of healthy individuals, reflecting a certain level of activation in response to environmental antigenic challenge (Hemler et al., 1984; Zola et al., 1989). In humans, a large proportion of CD4 memory T cells also express intermediate levels of CD25 (Triplett et al., 2012). Our findings may indicate that the exercise mobilization of activated/memory CD4 T cells can be downregulated by immersion in water at thermoneutral or lower temperatures.

Exercise also induced a transient shift in blood monocyte composition. While the early response to exercise (post-ex and post-im) was marked by reduced classical monocytes and increased intermediate ones, the later response (24 h post-ex), after the additional bout of exercise (performance test), was the opposite, and resulted in a lower percentage of non-classical monocytes. This pattern of monocyte subsets mobilization by exercise that has been previously demonstrated (Steppich et al., 2000; Slusher, Zúñiga and Acevedo, 2018), was affected by thermoneutral and hot-water immersions. In both 28 and 38°C water, the percentages of non-classical monocytes were likely higher post-recovery, compared to control. This finding means that post-exercise recovery by hot water-immersion not only reduced monocyte mobilization, but also increased the proportion of non-classical monocytes among these cells. To date, only one study investigated the effect of water immersion on monocyte response to exercise (Jajtner et al., 2014). After a simulated lower body resistance training, the expression of the complement receptor-3 (CR3) was increased in blood CD14^+^ monocytes, and cold-water immersion (10 min, 10^o^–12°C) likely attenuated this response. However, the study did not consider the different monocyte subsets. Recently, the effects of passive heating on inflammatory markers were investigated (Hoekstra et al., 2021). Participants (sedentary men) were immersed up to the neck, in 39°C water for 1 h. In the control situation the same participants remained in rest, for 1 h, in an environmental chamber (27°C, 40% humidity). Immersion in hot water for 1 hour resulted in the elevation of rectal temperature and, like our findings, was also associated with a greater mobilization of intermediate and non-classical monocytes, without effects on the classical subset. Non-classical monocytes are the primary producers of tumor necrosis factor and IL-1β (Wong et al., 2011; Mukherjee et al., 2015). These cells patrol the blood vessels’ endothelium and are thought to be involved in tissue surveillance and repair (Auffray et al., 2007; Cros et al., 2010). It is hypothesized that the mobilization of CD16^+^ monocytes from the marginal pool of cells during the stress of exercise may serve to provide a large population of active cells for defense at sites of injury and infection (Steppich et al., 2000). These cells can also have a role in the restoration of muscle homeostasis after exercise, by facilitating the clearance of debris from the damaged skeletal muscle (Chazaud et al., 2009). Moreover, they can be involved in the regulation of vascular health and function after acute exercise (Radom-Aizik et al., 2014). So, the increased mobilization of non-classical monocytes by thermoneutral and hot-water immersion may potentiate the effects of exercise on skeletal muscle biology. Our data also suggest that these effects are dependent on water temperature. Therefore, water temperature appears to play a role in the leukocyte response following exhaustive exercise.

Despite the alterations observed on blood leukocyte response to exercise by water immersion, we found no effect of the interventions on serum CK and AST. We measured these two enzymes because intensive muscle activity during exercise can accelerate fascia permeability, thus leading to the leakage of these enzymes (Koutedakis et al., 1993; Flynn et al., 1994). Damage to proteins and membranes within exercised muscle result in increased inflammatory mediators, such as prostaglandins and cytokines, which promote the migration of immune cells to the muscle (Smith et al., 2000; Allen, Sun and Woods, 2015). Thus, modification in the dynamics of these markers would give us clues about the modulation of muscle derived signals that could explain modifications on blood leukocyte populations and subpopulations. Although CK and AST leakage was not affected by water immersion in this study, that could be due to the kinetics of these markers in response to exercise. It does not exclude the possibility that recovery using water immersion modulates the production/liberation of other mediators that could account for the results obtained. This, in fact, is the main limitation of this study: the lack of measurement of inflammatory mediators, such as IL-6 and IL-1 or chemokines that could help us to understand which factors would be mediating the leukocyte response to water immersion recovery. Also, to test whether blood leukocyte modulation by water immersion would impact local muscle cell infiltration will be necessary to comprehend how exercise recovery by water immersion affects muscle biology. These evaluations, associated with fatigue, local muscle pain and swelling and muscular function, would allow researchers to draw a more complete picture of inflammation modulation by water immersion on skeletal muscle function.

Although the physiological significance of post-exercise inflammation on muscle function and biology has been recognized, reduced muscle capacity and exercise performance has been also associated with inflammation. We have previously demonstrated that cold-water immersion likely and thermoneutral-water immersion possibly improved the recovery of running performance after the exhaustive exercise protocol used in the present study, while the effectiveness of recovery by hot-water immersion was unclear (De Paula et al., 2018). Based on this, and because immersion in cold and hot water are associated with distinct vascular responses, we hypothesized that cold-water but not hot-water immersion would attenuate the exercise-induced leukocytosis. Our initial hypothesis was, therefore, refuted. And our findings indicate that the modification of the leukocyte response to exercise by water-immersion recovery does not parallel the effects of water immersion observed on running performance. One possible explanation for this dissociation can be the large time interval between the end of the immersion and the time of the running performance assessment (4 h, approximately). During this interval other factors associated with running performance, besides inflammation, such as internal temperature and glycogen resynthesis, may have had their recovery accelerated by immersion. As we previously showed, the recovery of rectal temperature was accelerated by cold water immersion (De Paula et al., 2018).

The main contribution of this study to the field is the effect of hot-water immersion on the exercise-induced leukocyte dynamics. The number of studies that investigated the effects of hot-water immersion on the acute inflammatory response to exercise is limited, compared to cold or temperate thermoneutral water immersions, and they are mainly focused on eccentric exercise models. This can be due to the idea that in the post-exercise acute phase or during the first inflammatory phase, heat may reinforce the inflammatory response (Hotfiel et al., 2018). However, post-exercise recovery can benefit from heat because circulation and tissue perfusion are essential for tissue healing (Whitney, 1990). In this line, our findings suggest that hot water immersion can control the magnitude of the inflammatory response to the exercise, as leukocyte mobilization was likely reduced by the intervention. At the same time, hot water immersion regulates the qualitative profile of the response, by increasing the mobilization of different monocyte subtypes. Also, different from most of the studies that used hot water, we used an exercise protocol that caused not only neuromuscular but also cardiovascular stress, as previously demonstrated (De Oliveira Ottone et al., 2014; De Paula et al., 2018). This resembles the daily activity of different sports modalities, where both resistance and endurance training are performed concurrently, and training sessions occurs more than once a day. It is worth mentioning that the cardiovascular and thermoregulatory response to the exercise protocol we used were the same on all occasions (De Oliveira Ottone et al., 2014; De Paula et al., 2018) and thus, the observed effects were induced in response to the different recovery strategies used.

## Data Availability

The raw data supporting the conclusions of this article will be made available by the authors, without undue reservation.
