# Corrigendum: Modulation of leukocyte subsets mobilization in response to exercise by water immersion recovery

**DOI:** 10.3389/fphys.2022.1107414

**Published:** 2022-12-07

**Authors:** Vinícius de Oliveira Ottone, Fabrício De Paula, Paula Fernandes Aguiar Brozinga, Mariana Aguiar de Matos, Tamiris Campos Duarte, Karine Beatriz Costa, Bruna Caroline Chaves Garcia, Thyago José Silva, Flavio De Castro Magalhães, Cândido Celso Coimbra, Elizabethe Adriana Esteves, Kelerson Mauro de Castro Pinto, Fabiano Trigueiro Amorim, Etel Rocha-Vieira

**Affiliations:** ^1^ Exercise Biology and Immunometabolism Laboratory, Centro Integrado de Pós-graduação e Pesquisa em Saúde, Graduate Program in Physiological Sciences, Universidade Federal dos Vales do Jequitinhonha e Mucuri, Diamantina, Brazil; ^2^ Faculty of Medicine, Universidade Federal dos Vales do Jequitinhonha e Mucuri, Diamantina, Brazil; ^3^ Faculdade de Sete Lagoas, Sete Lagoas, Brazil; ^4^ Graduate Program on Health Sciences, Universidade Federal dos Vales do Jequitinhonha e Mucuri, Diamantina, Brazil; ^5^ Departament of Physical Education, Faculty of Biological and Health Sciences, Universidade Federal dos Vales do Jequitinhonha e Mucuri, Diamantina, Brazil; ^6^ Departament of Physiology and Biophysics, Faculty of Biological Sciences, Universidade Federal de Minas Gerais, Belo Horizonte, Brazil; ^7^ Departament of Nutrition, Faculty of Biological and Health Sciences, Universidade Federal dos Vales do Jequitinhonha e Mucuri, Diamantina, Brazil; ^8^ School of Physical Education, Universidade Federal de Ouro Preto, Ouro Preto, Brazil; ^9^ Exercise Physiology Laboratory, Department of Health, Exercise and Sport Science, University of New Mexico, Albuquerque, NM, United States

**Keywords:** hot-water immersion, non-classical monocytes, acute inflammation, CD25, lymphocyte, endurance exercise, resistance exercise

In the published article, there was an error in the **Funding** statement. This work was funded by two different grants from FAPEMIG (CDS-APQ 00908-08 and APQ-00581-11), although just one was mentioned (CDS-APQ 00908-08). The correct Funding statement appears below.

“This work was supported by Coordenação de Aperfeiçoamento de Pessoal de Nível Superior (CAPES) (PNPD-2455/2011 and Finance code 001), Fundação de Amparo a Pesquisa do Estado de Minas Gerais (FAPEMIG) (CDS-APQ 00908-08 and APQ-00581-11), and Conselho Nacional de Desenvolvimento Científico e Tecnológico (CNPq) (445096/2014-4) funding agencies”.

The authors apologize for this error and state that this does not change the scientific conclusions of the article in any way. The original article has been updated.

