# Supplementary material for: Modulation of Leukocyte Subsets Mobilization in Response to Exercise by Water Immersion Recovery
Source: Front Physiol. 2022 Aug 16;13:867362. doi: 10.3389/fphys.2022.867362 (PMC9425101; doi:10.3389/fphys.2022.867362)
Supplement: Supplementary file 1 [file DataSheet1.PDF]

Supplementary Material

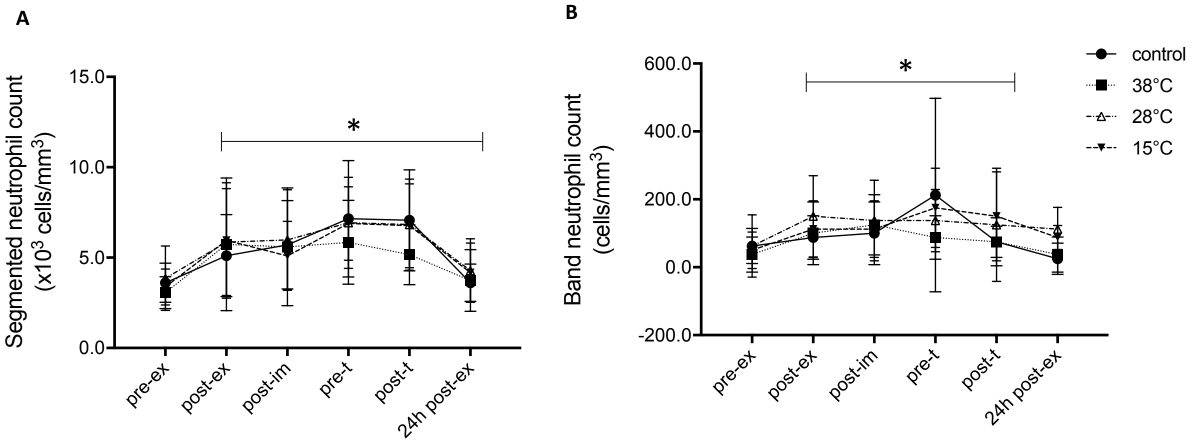

Supplementary Figure 1. Effect of post-exercise recovery by water immersion on segmented (**A**) and band neutrophil count (**B**). *P < 0.05, main time effect, compared to pre-ex, mixed effects model, followed by Dunnett's multiple comparison test. Pre-ex – before exercise, post-ex – immediately after exercise session, post-im - immediately post immersion, pre-t – immediately before performance test, post-t – immediately after performance tests, 24 h post-ex – 24 hours after the exercise session.

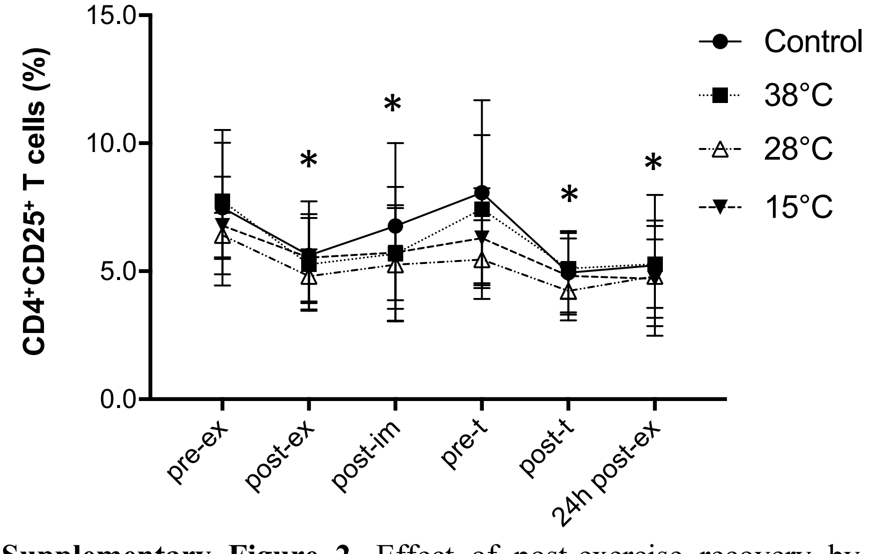

Supplementary Figure 2. Effect of post-exercise recovery by water immersion on the percentage of $CD4^{+}CD25^{+}$ T cells. *P < 0.05, main time effect, compared to pre-ex, mixed effects model, followed by Dunnett's multiple comparison test. Pre-ex – before exercise, post-ex – immediately after exercise session, post-im - immediately post immersion, pre-t – immediately before performance test, post-t – immediately after performance tests, 24 h post-ex – 24 hours after the exercise session.
